# RESEARCH AT THE INTERSECTION OF TECHNOLOGY AND AGING

**DOI:** 10.1093/geroni/igac059.1657

**Published:** 2022-12-20

**Authors:** Jodi McDaniel

## Abstract

New and emerging technologies offer opportunities for the delivery of wide-ranging, adaptable interventions to improve quality of life of older adults. Novel technologies can also improve the quality of measurement methods used in aging research. In this symposium five presentations will describe advanced technologies to promote health and function in older adults. The first will discuss the Urban Aging Residents Coalition (UARC), an organization founded in partnership with an African American older adult community leader to address engaging urban older adults with technology. A primary goal of UARC is to prevent social isolation and promote mental wellness through education and computer literacy. The second presentation will describe a project testing socially assistive robots (SARs) to complement personnel resources in older adults with cognitive impairment in long term care facilities. Study participants were successfully engaged in a participatory design involving repeated sessions with SARs that resulted in prototype refinement. The third presentation will describe current applications of patient communication technologies in acute-critical care settings with a focus on the user experience among older adult patients. The fourth will report on the implementation of an ongoing, in-home Smarthealth technology intervention for two older adult family caregivers of persons with dementia. Study findings showed the intervention improved self-awareness of emotional care and reactions to care recipients. The final presentation will explain an advanced wound measurement system using artificial intelligence to track healing progress in clinical research. The presentations will highlight technologies to support healthy aging and discuss implication for practice, policy, and research.

